# Study on the Photoluminescent and Thermal Properties of Zinc Complexes with a N_6_O_4_ Macrocyclic Ligand

**DOI:** 10.3390/molecules23071735

**Published:** 2018-07-16

**Authors:** Xingyong Xue, Qijun Wang, Fusen Mai, Xing Liang, Yichen Huang, Jiahe Li, Yanling Zhou, Dengfeng Yang, Zhen Ma

**Affiliations:** 1 School of Chemistry and Chemical Engineering, Guangxi University, Nanning 530004, China; xingyongxue@126.com (X.X.); dennis1992@live.cn (Q.W.); maifusen@163.com (F.M.); liangxing0123@163.com (X.L.); c-lf2@126.com (Y.H.); lijiahe006@gmail.com (J.L.); zyl8289@126.com (Y.Z.); 2 Guangxi Colleges and Universities Key Laboratory of Applied Chemistry Technology and Resource Development, Nanning 530004, China; 3 Guangxi Academy of Sciences, Guangxi Beibu Gulf Marine Research Center, Guangxi Key Laboratory of Marine Natural Products and Combinatorial Biosynthesis Chemistry, Nanning 530007, China; 4 Centro de Química Estrutural, Complexo I, Instituto Superior Técnico, Universidade de Lisboa, Av. Rovisco Pais, 1049-001 Lisbon, Portugal

**Keywords:** macrocyclic ligand, zinc complexes, photoluminescent properties, thermal properties

## Abstract

Reactions between a N_6_O_4_ macrocyclic ligand (L^1^) and several Zn(II) salts (trifluoromethane sulfonate, *p*-toluenesulfonate, acetate, benzoate, *o*-, *m*- or *p*-hydroxybenzoate) led to the formation of seven complexes, [Zn_2_L^1^ (DMSO)_4_](OSO_2_CF_3_)_4_ (**1**), [Zn_2_(*p*-OSO_2_PhCH_3_)_4_L^1^] (**2**), [Zn_2_(OCOCH_3_)_4_L^1^] (**3**), [Zn_2_(OCOPh)_4_L^1^] (**4**), [Zn_2_(*o*-OCOPhOH)_4_L^1^] (**5**), [Zn_2_(*m*-OCOPhOH)_4_ L^1^] (**6**) and [Zn_2_(*p*-OCOPhOH)_4_ L^1^] (**7**), which were characterized by elemental analysis, ^1^H-NMR, ^13^C-NMR, IR, fluorescence spectroscopies and single crystal X-ray diffraction. In **1**, the Zn atom is pentacoordinated with a N_3_O_2_ irregular trigonal bipyramidal coordination environment, like the geometries in compounds **3**–**7**, whereas in structure **2** the metal atom is envisaged as possessing a distorted N_3_O_3_ octahedronal environment. All the compounds show interesting photoluminescent properties in solid states and solutions in DMF and DMSO, which are reported along with their TG-DTA thermal decomposition processes, UV-vis absorption spectroscopy and fluorescence quantum yields in DMF and DMSO.

## 1. Introduction

Macrocyclic and macrobicyclic compounds are attracting increasing attention owing to their interesting structures, properties and important roles in coordination chemistry and many other fields, such as material science and medicinal chemistry [1,2,3,4,5,6,7,8,9,10,11,12,13,14,15,16,17]. With oxygen, nitrogen and/or sulfur donor atoms, these cyclic compounds can exhibit novel electrochemical, photoluminescent and catalytic properties [18,19,20,21,22,23,24,25,26,27]. It is well known that metal cryptates show interesting photoluminescent properties when some special chemical groups such as heterocycles [28,29,30,31,32,33,34] or phenols [35,36] are attached to strands of ligands. Macrocyclic and macrobicyclic compounds can encapsulate metal ions in their cavity and shield these metal ions from interaction with solvents and thus prevent radiationless luminescence emission deactivation processes and increase the kinetic and thermodynamic stability of the compounds in solution by the cryptate effect [37]. For example, when some lanthanide ions, such as Eu^3+^ and Tb^3+^, form cryptates with a cryptand, a strong emission band with long lifetime is observed. Interestingly, the introduction of a second Zn^2+^ or Cd^2+^ ion into the mononuclear Eu (III) cryptate resulted in a threefold increase of the luminescent intensity and a small increase in the molar absorption coefficients [37].

**Scheme 1:**
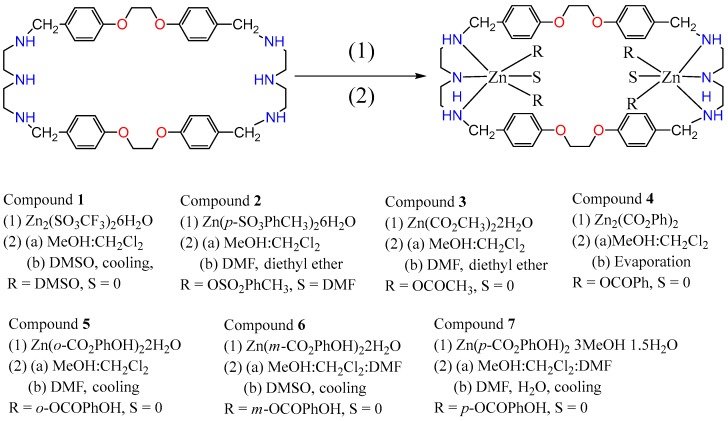
Synthetic routes for compounds **1**–**7**.

The current work aims to prepare metal complexes constructed from polyaza-ligands with organic sulfonate or organic carboxylic groups as auxiliary ligands and to investigate their coordination and photoluminescent behaviors. Thus, herein, we report the syntheses of a series of new zinc compounds [Zn_2_L^1^ (DMSO)_4_](SO_3_CF_3_)_4_ (**1**) and [Zn_2_L^1^L^2^] (**2**–**7**) (where L^1^ = 1,4,19,22,25,40-hexaaza-10,13,31,34-tetraoxa-6,14,27,35(1,4)-tetrabenzenacyclopentacontane; L^2^ = OSO_2_PhCH_3_^−^ and DMF, OCOCH_3_^−^, OCOPh^−^, *o*-OCOPhOH^−^, *m*-OCOPhOH^−^, and *p*-OCOPhOH^−^), which were assembled from the N_6_O_4_ hexadentate ligand and zinc salts (trifluoromethane sulfonate, *p*-toluenesulfonate, acetate, benzoate and *o*-, *m*- or *p*-benzoate derivatives) (Scheme 1). Their solid state structures (as shown by single crystal X-ray diffraction) display five- or six-coordinated environments, where each Zn^2+^ is coordinated by three N atoms of the macrocyclic ligand and two oxygen atoms from the auxiliary ligands, or a third oxygen atom from a solvent. Their fluorescent and thermal properties were also studied.

## 2. Results and Discussion

### 2.1. Elucidation of the Structures of the Compounds

The seven complexes were isolated as white solids which were characterized (see Materials and Methods) by elemental analysis, ^1^H-NMR (e.g., the protons of the –OH group in **5**, **6** and **7** resonate at *δ* 13.48, 9.32, 9.77, respectively), ^13^C-NMR (the carbons bearing hydroxyls appear at *δ* 162.32, 157.48 and 162.33 and the carboxylate carbons of the corresponding ligands at *δ* 170.8–173.2), IR and fluorescence spectroscopies, as well as by X-ray diffraction. The sequence of the ^1^H-NMR data is in accordance with that of the respective zinc compounds with 4′-Ph-terpyridine ligands (*δ* 13.22, 9.34, and 9.79, whereas the carbons with hydroxyls appear at *δ* 161.15, 156.86 and 159.79 and the carboxylate carbons of the corresponding ligands at *δ* 170.8–173.1 in the ^13^C-NMR spectra) [38]. Crystals of all the complexes suitable for X-ray analysis were obtained upon crystallization from DMSO (**1**, **6**, heating and cooling), DMF (**2**, **3**, diffusion of diethyl ether; **5**, heating and cooling), DMF and H_2_O (**7**, heating and cooling) or slow evaporation of the mother liquor solution (compound **4**). Crystal data and data collection details are reported in Table 1.

Compound **1** crystallizes with two molecules of DMSO, **2** with four DMFs, one 1,4-dioxane and one water, **3** with two DMFs and four waters, **4** with two methanols and three waters, **5** with two methanols and two waters, **6** with four DMSOs and **7** with two waters, respectively.

**Figure 1:**
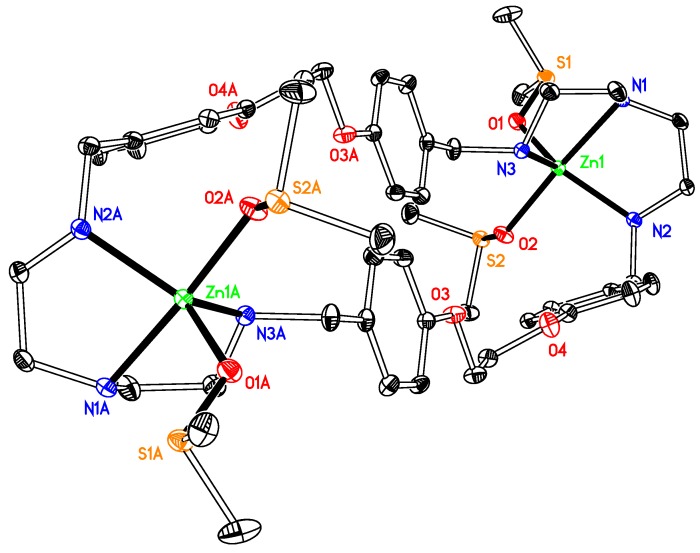
Thermal ellipsoid plot, drawn at the 50% probability level, of [Zn_2_(OS(CH_3_)_2_)_4_L^1^]^4+^ (**1**) with atomic numbering scheme. All H atoms and the counterions are omitted for clarity. Symmetry codes: A 1 − x, −y, −z. Selected bond lengths (Å) and angles (°): Zn1-N1 2.1484(18), Zn1-N2 2.084(2), Zn1-N3 2.0922(18), Zn1-O1 2.0399(17), Zn1-O2 2.0556(16); N1-Zn1-N2 83.01(6), N2-Zn1-N3118.58(7), N1-Zn1-N3 84.43(7), O1-Zn1-O2 84.80(7).

[Zn_2_L^1^ (DMSO)_4_](OSO_2_CF_3_)_4_ **1** is a dinuclear complex (Figure 1). The zinc ion is coordinated with five atoms, three nitrogen atoms from the macrocyclic ligand, two oxygen atoms from two DMSOs, involved in a distorted ZnN_3_O_2_ irregular trigonal bipyramidal geometry with one coordinated oxygen atom of DMSO and the bridgehead nitrogen atom occupying the apical positions [bond angles range from 83.01(7) to 99.09(7)° between the apical atoms and the equatorial positions]. This type of geometry is that usually reported for related Zn(II) compounds, e.g., [Zn(OSO_2_CF_3_)_2_L (OH)] (L = 4′-phenyl-2,2′:6′,2′′-terpyridyl) [38], [Zn_2_(μ-O_2_SO_2_)_2_ L (CH_3_OH)_2_] (L = 4′-phenyl-2,2′:6′,2′′-terpyridyl) [39], or [Zn(OSO_2_CF_3_)_2_ L (H_2_O)] [L = 12,12-dimethyl-7,10,14,17-tetraoxa-1(3,6),3(2,5),2(2,6)-tripyridina-11,13(1,4)-dibenzenacycloicosaphane] [40]. Several intermolecular hydrogen bonds exist in the structure, and three of them are worth to be mentioned. They involve one hydrogen atom at the coordinated nitrogen atom (N3) of the ligand and one sulfur atom (S1) of the coordinated DMSO molecule and one oxygen atom (O5) from a neighbouring DMSO molecule, or one hydrogen atom at the coordinated nitrogen atom (N2) of the ligand and one oxygen atom (O11) at one CF_3_SO_3_^−^counterion, along with several others involving the other oxygen atoms with hydrogen atoms at the carbon atoms of aryl groups of the ligand. In its structure, due to the position and orientation of the DMSO molecules in **1**, the Zn complex molecules are too far apart for any convenient π-π interaction. However, three π-ring interactions (X–H···Cg) exist in the structure, with the values of 3.772(2), 3.672(4) and 3.672(4) Å, respectively.

**Figure 2:**
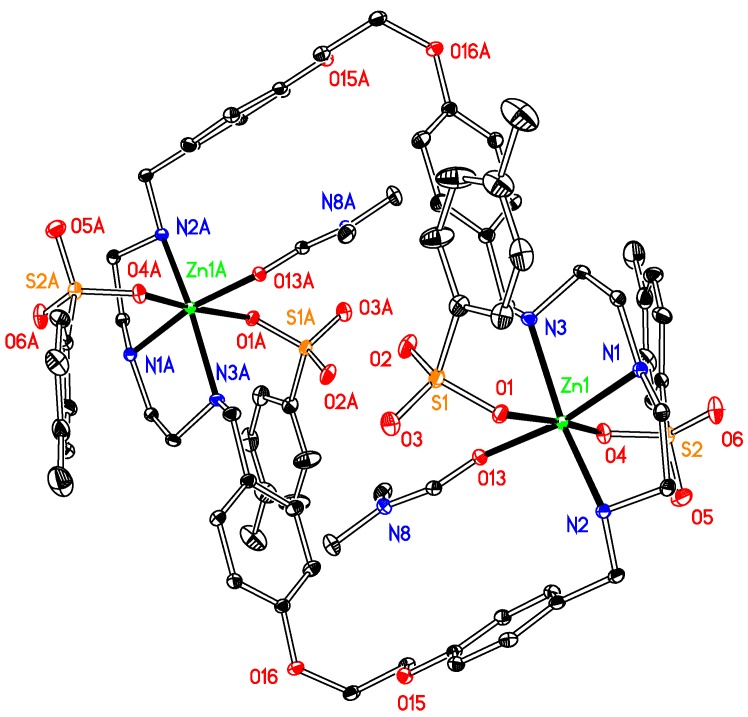
Thermal ellipsoid plot, drawn at the 50% probability level, of [Zn_2_(*p*-OSO_2_PhCH_3_)_4_L^1^] (**2**) with atomic numbering scheme. All H atoms, along with DMF, C_4_H_8_O_2_, are omitted for clarity. Symmetry codes: A 1 − x, 1 − y, −z. Selected bond lengths (Å) and angles (°): Zn1-N1 2.122(18), Zn1–N2 2.168(2), Zn1-N3 2.182(2), Zn1–O1 2.4094(19), Zn1–O4 2.086(2); Zn1–O13 2.0390(18); N1–Zn1–N2 82.36(8), N2–Zn1–N3 158.21(8), N1–Zn1–O13 169.81(8), O1–Zn1–O4 174.32(7).

The asymmetric unit of compound **2** (Figure 2) comprises two half molecules because of the symmetry of the compound. The dinuclear complex crystallized in a centrosymmetric space group *P*-1. Each zinc ion is coordinated by three N atoms of the macrocyclic ligand and three oxygen atoms from two SO_3_PhCH_3_^−^ ions and one DMF, therefore forming an irregular N_3_O_3_ octahedronal coordination environment. The average bond length for Zn–N is 2.157(2) Å, which is 2.178 Å for Zn–O. The structure presents no intermolecular π-π interactions but twelve π-ring (X–H...Cg) interactions between oxygen atoms at the sulfonates and their neighbouring rings with an atom-centroid distances (X···Cg) in a range of 3.553–4.23 Å. Further stabilization, by several intermolecular hydrogen bonds, can also be found, involving the oxygen atoms at sulfonate, 1,4-dioxane or DMF molecules and the hydrogens at carbon atoms of the ligand, although no classic hydrogen bond is present in the structure.

**Figure 3:**
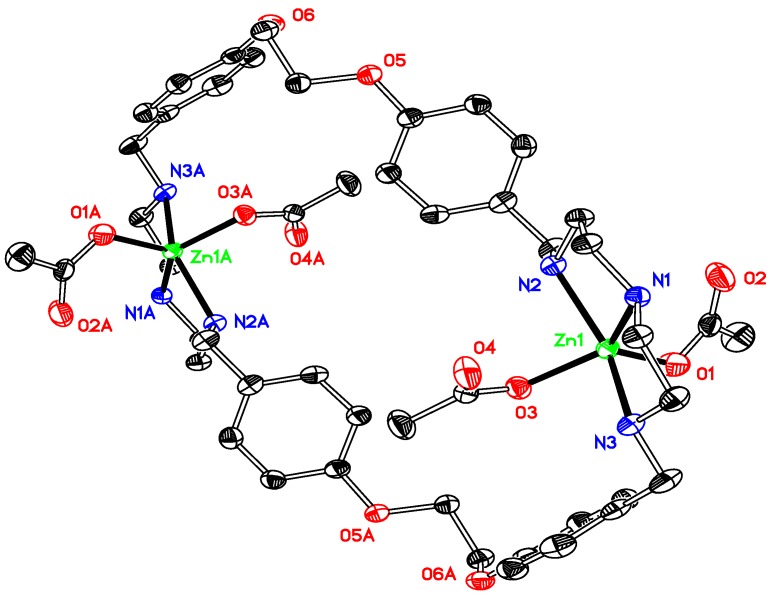
Thermal ellipsoid plot, drawn at the 50% probability level, of [Zn_2_(OCOCH_3_)_4_L^1^] (**3**) with atomic numbering scheme. All H atoms, along with DMFs and waters are omitted for clarity. Symmetry codes: A 1 − x, −1 − y, −z. Selected bond lengths (Å) and angles (°): Zn1–N1 2.0968(14), Zn1–N2 2.218(14), Zn1-N3 2.2024(14), Zn1-O1 1.9456(13), Zn1-O3 2.0184(13); N1–Zn1–N2 80.46(5), N2–Zn1–N3 151.42(5), N1–Zn1–N3 81.50(5), O1–Zn1–O3 100.98(6).

**Figure 4:**
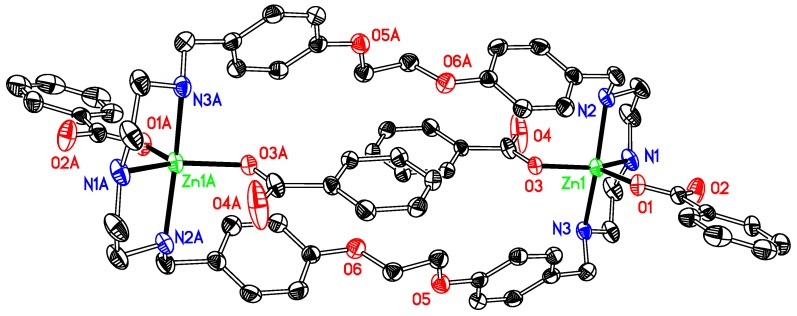
Thermal ellipsoid plot, drawn at the 30% probability level, of [Zn_2_(OCOPh)_4_L^1^] (**4**) with atomic numbering scheme. All H atoms, along with the solvents are omitted for clarity. Symmetry codes: A 1 − x, −y, −z. Selected bond lengths (Å) and angles (°): Zn1–N1 2.116(3), Zn1–N2 2.177(3), Zn1–N3 2.237(3), Zn1–O1 1.991(2), Zn1–O3 1.979(2); N1–Zn1–N2 80.80(13), N1–Zn1–N3 79.70(12), N2–Zn1–N3 156.08(12), O1–Zn1–O3 109.48(11).

**Figure 5:**
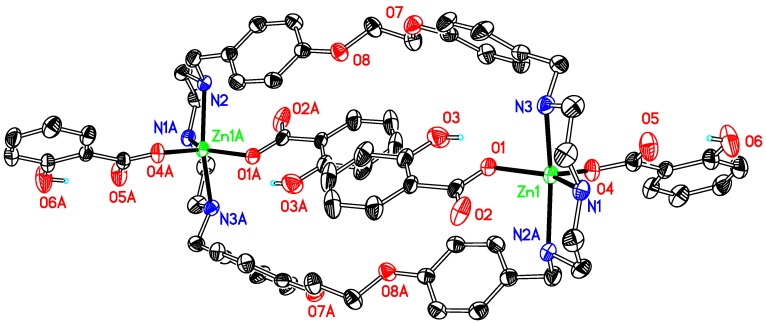
Thermal ellipsoid plot, drawn at the 50% probability level, of [Zn_2_(*o*-OCOPhOH)_4_L^1^] (**5**) with atomic numbering scheme. All H atoms, along with the solvents are omitted for clarity. Symmetry codes: A −x, 1 − y, 1 − z. Selected bond lengths (Å) and angles (°): Zn1–N1 2.096(3), Zn1–N2A 2.260(3), Zn1–N3 2.236(3), Zn1-O1 1.995(3), Zn1–O4 1.980(3); N1–Zn1–N2A 80.21(12), N2A–Zn1–N3 155.18(13), N1–Zn1–N3 79.96(10), O1–Zn1–O4 105.97(13).

**Figure 6:**
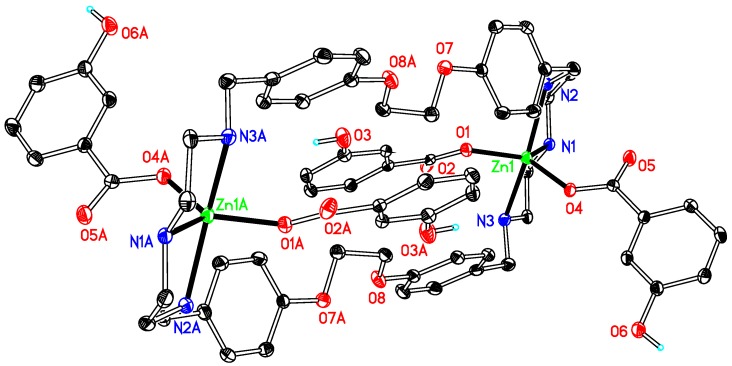
Thermal ellipsoid plot, drawn at the 50% probability level, of [Zn_2_(*m*-OCOPhOH)_4_L^1^] (**6**) with atomic numbering scheme. All H atoms, along with the solvents are omitted for clarity. Symmetry codes: A 2 − x, 1 − y, 1 − z. Selected bond lengths (Å) and angles (°): Zn1–N1 2.063(3), Zn1–N2 2.298(2), Zn1–N3 2.275(2), Zn1–O1 1.994(2), Zn1–O4 1.953(2); N1–Zn1–N2 80.38(10), N2–Zn1–N3 158.43(10), N1–Zn1-N3 80.26(10), O1–Zn1–O4 113.13(9).

**Figure 7:**
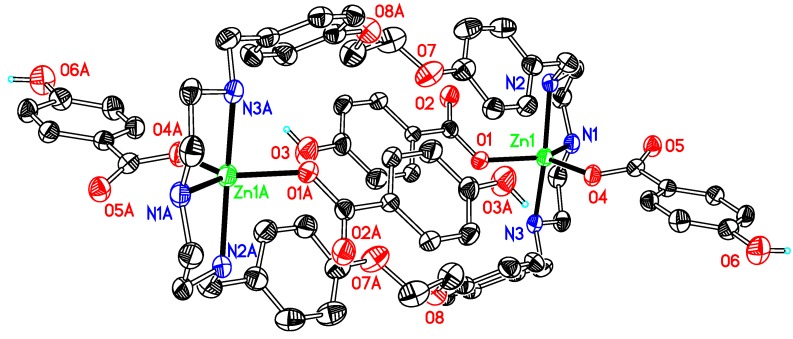
Thermal ellipsoid plot, drawn at the 30% probability level, of [Zn_2_(*p*-OCOPhOH)_4_L^1^] (**7**) with atomic numbering scheme. All H atoms, along with the solvents are omitted for clarity. Symmetry codes: A −x, −y, −z. Selected bond lengths (Å) and angles (°): Zn1–N1 2.096(3), Zn1–N2 2.202(3), Zn1–N3 2.242(4), Zn1–O1 1.977(3), Zn1–O4 1.971(3); N1–Zn1–N2 81.27(14), N2–Zn1–N3 157.98(12), N–Zn1–N3 80.23(13), O1–Zn1–O4 118.13(12).

Like **1**, compounds **3**, **4**, **5**, **6** and **7** (Figure 3, Figure 4, Figure 5, Figure 6 and Figure 7) are also dinuclear species that crystallize in centrosymmetric space groups *P*21/*c* (**3** and **4**) or *P*-1 (**5**, **6** and **7**) and the difference is that the auxiliary ligand and DMSO are substituted by acetate, benzoate, *o*-, *m*-, or *p*-hydroxybenzoate, respectively. The asymmetric units of the compounds present half of the molecule in **3**, **5** and **6**, two half molecules in **4** and **7**, respectively, because of the symmetry of the molecules. The coordination environment around the zinc ion in these compounds displays an irregular trigonal bipyramidal geometry, as that in complex **1** and other related compounds [28,29], which results from the coordination of zinc by three nitrogen atoms from a macrocyclic ligand and two oxygen atoms from the auxiliary ligands in a monodentate mode. The average contact between the central metal ion and the oxygen atoms (Zn–O) is 1.982 Å in **3** and 1.985 Å in **4**, both of them have no hydroxyl group at their auxiliary ligands, being nearly equal to that in **5** (1.988 Å) and longer than those in **6** (1.974 Å) and **7** (1.974 Å). This is indicative that the hydroxyl substituent has a shortening effect on the Zn–O bond lengths and the phenomenon is contrary to the results observed in a series zinc benzoate compounds, obtained by the reaction of zinc benzoate salts and a terpyridine ligand [38]. However, these bond lengths do not follow the sequence of pK_a_ values of the conjugated acids of the anions, the latter being 2.98 (for *o*-), 4.08 (for *m*-), 4.17 (for benzoic acid) and 4.58 (for *p*-hydroxybenzoic acid). Such bond lengths are not only affected by the basicity of the anions but also by other factors, including hydrogen bonds, or even spatial environments determined by the positions of the hydroxyl groups at the phenyl rings [38].

It is worth mentioning the distances of the metal-metal and bridge-head nitrogen atoms of the ligands in the macrocyclic compounds of this work since these values show that the structures of the ligand have changed according to the environments of the metal ions and the auxiliary ligands. The distances between the two metal ions in these compounds are 16.686 Å for **1**, 10.9858(6) and 10.7890(6) Å for **2**, 11.8357(6) Å for **3**, 14.449(1) and 14.524(1) Å for **4**, 14.2647(8) Å for **5**, 14.6245(9) Å for **6** and 14.727(8) and 14.490(1) Å for **7**. The distances between the two bridge-head nitrogen atoms are 17.725(4) Å for **1**, 14.416(4) and 14.427(4) Å for **2**, 14.554(3) Å for **3**, 18.446(7) and 18.570(7) Å for **4**, 17.957(6) Å for **5**, 18.662(5) Å for **6** and 18.842(6) and 18.509(8) Å for **7**, respectively. These data reflect the structures of the ligand in these compounds, concerning the three parts of it, including two N3 fragments (form two N3 planes) and four oxygen atoms at the two strands (form the third plane) stretch or fold in these compounds. The principal of the structures is that the longer distances between the metal ions and the bridge-head nitrogen atoms, the more stretching and less folding of the ligand. The shortest distances between metal ions and bridge-head nitrogen atoms are in **2**, and the ligand in this compound forms a parallelogram structure at O16, O16A, N2 and N2A in one molecule and O17, O17A, N5 and N5A in the other with the two aryl groups at one strand of the ligand parallel to those at the other one, with the side lengths being 8.940(3), 10.756(3), 8.940(3) and 10.756(3) Å for one molecule and 8.919(3), 10.734(3), 8.919(3) and 10.734(3) Å for the other. In the structures of the compounds (**4**, **6** and **7**) with the distance of the two bridge-head nitrogen atoms over 18 Å, the N3 fragment is coplane with the plane formed by the four oxygen atoms at the two strands of the ligand.

The dihedral angles between the two planes formed by the N3 part at the end of the ligand and four oxygen atoms at the two strands of the ligand can be also used to illustrate the conformations of the ligand in these complexes whether the ligand is with a chair or plane conformation, which are 43.50(7)° for **1**, 5.75(14)° and 8.89(4)° for **2**, 31.52(5)° for **3**, 26.06(18)° and 17.24(19)° for **4**, 29.73(20)° for **5**, 1.97(8)° for **6** and 9.08(27)° and 5.72(30)° for **7**, respectively. A rule is set up to show clearly the conformations of the ligand in these structures and that is: the ligand is with a chair conformation when the dihedral angle is greater than 30°, a half-chair when it is in the range of 10–30° and a plane conformation when smaller than 10°. According to these data and the rule, the ligand is with a chair conformation in the structures of **1** and **3**, a half-chair conformation in **4** and **5**, whereas a plane conformation in **2**, **6** and **7**. Several hydrogen bonds and/or π-ring interactions exist in each structure of the complexes. The structure of **3** presents just hydrogen bonds and no π-ring interaction is observed. The hydrogen bonds involve the hydrogen atoms at the coordinated nitrogen atoms (N1 and N3) of the ligand, two water molecules, three carbon atoms of the ligand and one DMF and several oxygen atoms from the water molecules, DMF and the auxiliary ligands in the range of 2.73–3.53 Å. There are several hydrogen bonds presenting in the structure of **4** in the range of 2.47–3.32 Å, involving the hydrogen atoms at the coordinated nitrogen atoms (N1, N2, N4 and N6) of the ligand, one water molecule, one methanol and two carbon atoms of the ligand and several neighbouring oxygen atoms from one water molecule and the auxiliary ligands. In **4**, several π-ring interactions are present (X–H···Cg are in the range of 3.58–4.09 Å) between several H atoms at the three –CH_2_– groups of the ligand and one aryl carbon atom of one auxiliary ligand and their neighbouring rings.

In the remaining structures (**5**–**7**), the range of hydrogen bond is 2.54–3.14 in **5**, 2.67–3.41 in **6** and 2.63–3.39 Å in **7**, involving the hydrogen atoms in coordinated nitrogen atoms in the ligand and the hydroxyl groups of the auxiliary ligands and the oxygen atoms at the carboxyl groups of the auxiliary ligands in **5**, the hydrogen atoms at the coordinated nitrogen atoms of the ligand, the hydroxyl groups of the auxiliary ligands, the carbon atoms of the ligand, auxiliary ligands and DMSO and the oxygen atoms at the ligand, the carboxyl and hydroxyl groups of the auxiliary ligands and DMSO in **6**, the hydrogen atoms at the nitrogen and carbon atoms of the ligand, the hydroxyl groups and aryl carbon atoms of the auxiliary ligands and water molecules and the oxygen atoms at the auxiliary ligands and one water molecule in **7**, respectively.

In the structure of **5**, there are three kinds of π-ring (X–H···Cg) interactions: one is between one hydrogen at O3 of the hydroxyl group of the auxiliary ligand and a neighbouring aryl ring of the ligand with an atom-centroid distance (X···Cg) of 3.3260 Å; the second one is between the hydrogen atom at one carbon atom (C13) of the ligand and one neighbouring aryl ring of the neighbouring ligand with the atom-centroid distances (X···Cg) of 3.6606; the third one is between a hydrogen atom at one –CH_2_– of the ligand and one neighbouring aryl group of a auxiliary ligand. In **6**, two π-ring interactions present (H–X···Cg being 3.4768 and 3.6463 Å) between two H atoms at one DMSO and their neighbouring aryl rings of the ligand and auxiliary ligand, which are in the range of 3.59–3.78 Å for X–H···Cg in **7**, between four hydrogen atoms of four –CH_2_– of the ligand and one hydrogen atom at one aryl group of one auxiliary ligand and five neighbouring aryl rings of the ligand or auxiliary ligands. One Y–X···Cg also exists in **7**, which involves one oxygen atom (O13) and one neighbouring aryl group of the ligand

### 2.2. TG-DTA Properties of the Complexes

**Figure 8:**
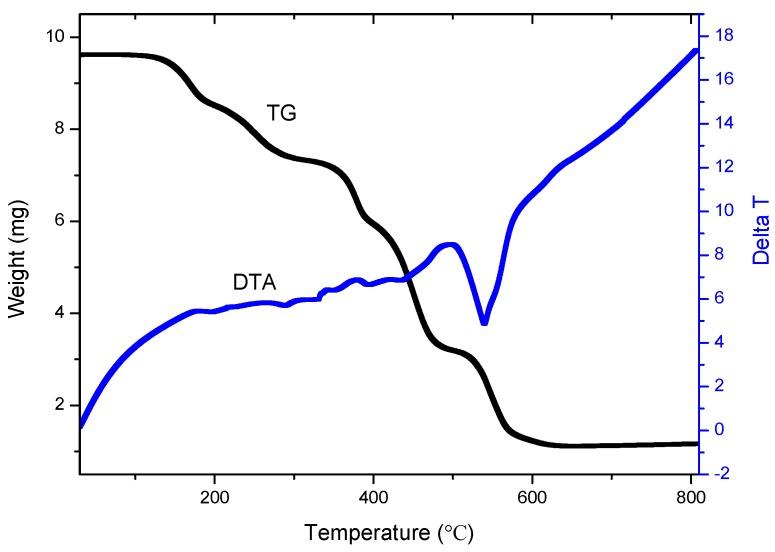
The TG-DTA curves of compound **1**.

Figure 8 shows the TG and DTA curves for complex **1**. The molecules display five major decomposition steps corresponding to loss of the solvent and four decomposition processes. The first loss is in the range of 120–200 °C (centered at 160 °C) and it concerns the loss of the uncoordinated DMSO molecules. The second loss concerns the loss of the coordinated DMSO molecules in the range of 200–325 °C (centered at 262 °C). The weight loss in the two steps is 24.0% (calc. 25.0%). The third decomposition occurs in the temperature range of 325–400 °C (centered at 362 °C) and corresponds to a 14.1% loss of weight. The fourth decomposition occurs in the temperature range of 400–499 °C (centered at 450 °C) and corresponds to a 24.5% loss of weight. The loss in the final decomposition process is 2.08 mg within the temperature range of 499–645 °C (centered at 573 °C) with the percentage of 21.6%. The final product is ZnO and its weight is 2.99 mg with the percentage of 11.6, which nearly matches with the calculated value of 8.7%. The higher percentage than that of calculated value indicates that a small amount of ZnSO_4_ formed in the decomposition process.

**Figure 9:**
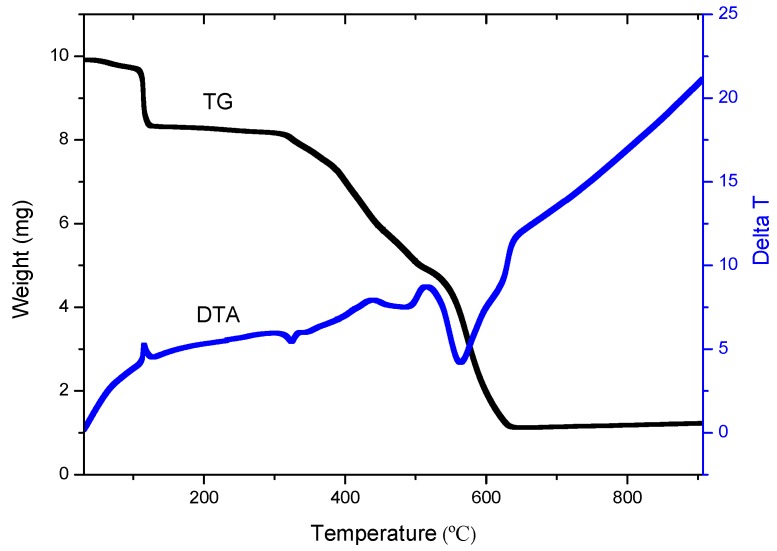
The TG-DTA curves of compound **2**.

The TG and DTA curves of **2** (Figure 9) have three major weight losses corresponding to a gradual loss of solvents in the compound, including water, 1,4-dioxane and DMF and two decomposition steps, in the range of 70–166, 308–516 and 516–644 °C, respectively. The loss for every step is 15.9, 34.6 and 38.1% (calc. 16.3% for first step), respectively. The final stable temperature in **2** is 644 °C, appears to lead to the formation of ZnO, with the percentage of 11.4%. The percentage is higher a little than that of calculated value (9.1%), indicating that a small amount of ZnSO_4_ also formed in the decomposition process. The TG and DTA curves of **3** give three major steps corresponding to loss of the solvents and two decomposition processes of the molecules (Figure S1). The first loss is in the range of 50–200 °C (centered at 125 °C) and it is a decomposition process of the solvents. The weight loss in this step is 16.2%. In the second loss, the temperature range for decomposition is 250–450 °C (centered at 300 °C) and it is a slow decomposition process with the 44.0% loss of weight. The loss in the final decomposition process is 7.12 mg within the temperature range of 450–680 °C (centered at 565 °C) with the percentage of 26.4%. The final result is ZnO and its weight is 3.334 mg with the percentage of 12.4%, which is matching with the calculated value 12.8%.

The TG-DTA properties of the benzoate complexes **4**, **5**, **6** and **7** were also studied and their decomposition processes are summarized in Table 2. The final product of thermal decomposition of the four complexes is also ZnO, which corresponds to 12.5% for **4**, 10.6% for **5**, 10.9% for **6** and 11.0% for **7**, complying with the theoretical value of 12.0% (**4**), 10.7% (**5**), 10.7% (**6**), and 10.6% (**7**), respectively.

The results show that the final stable temperatures and the decomposition processes depend greatly on the auxiliary ligands. By contrast those of **1** and **2** bearing the substituted sulfonate ligands, the final stable temperatures of complexes **3**–**4** with acetate or benzoate groups are high, but those of the benzoate complexes **5**–**7** with the hydroxyl group are low. Complex **3**, with the acetate as auxiliary ligand, displays the highest final state temperature (680 °C) among all seven compounds. Complex **6**, with the *meta*-OH substituent, is the benzoate complex with the lowest final state temperature (518 °C). With the hydroxyl substituent at the *para*-position, complex **7** displays the highest final state temperature (569 °C) in the three hydroxyl substituent compounds. All these sequences are different from the results obtained for Zn compounds with same counterions but a different ligand, 4′-phenyl-2,2′:6′,2′′-terpyridine [38]. The complex, with the hydroxyl substituent also at the *para*-position and 4′-phenyl-2,2′:6′,2″-terpyridine as the ligand, shows the lowest final state temperature (491 °C) in the three hydroxyl substituent complexes [38]. The result indicates that the differences in ligands, auxiliary ligands or intermolecular interactions, including hydrogen-bonds (which in the case of the *ortho*-OH complex 5 can be intramolecular) are expected to play the important roles in the decomposition processes.

### 2.3. Photoluminescent Properties

**Figure 10:**
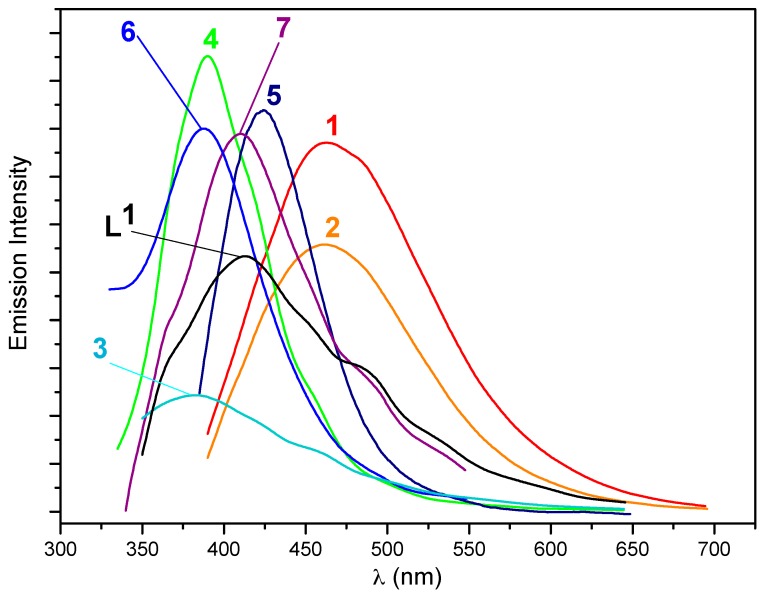
Solid state emission spectra of L^1^ and complexes **1**–**7** at room temperature (the determination of compound **4** was run with a small amount of samples, a few mg, and under conditions of the narrow slits of emission and excitation than other compounds). The peak of emission is 413 nm for L^1^, 463 (**1**), 462 (**2**), 383 (**3**), 390 (**4**), 425 (**5**), 388 (**6**), 411 (**7**), respectively.

The ligand and compounds **1**–**7** show interesting photoluminescent properties in the solid state at room temperature and their emission spectra and the wavelengths of excitation and emission are presented in Figure 10 and listed in Table 3. The ligand shows a strong peak as 413 nm when exited at 310 nm, which is assigned as intraligand charge transfer (ILCT or *n* → π* of the phenoxyl of the ligand). The sulfonate compounds **1** and **2** show very strong photoluminescent bands in their spectra. Compound **1**, excited at 340 nm, has one band in its emission spectra at ca. 463 nm, which is similar to the photoluminescent band in the emission spectrum of **2**, which appears at ca. 462 nm (excited at 360 nm). These results are indicative that the organic parts of the sulfonate or DMSO auxiliary ligands have no marked effects on the photoluminescence of the complexes. In comparison with the photoluminescence of L^1^ and the similar compounds with the substituted sulfonate as auxiliary ligands [38], the bands of **1** and **2** can tentatively be assigned to an ILCT.

For acetate complex **3**, one band is detected at 383 nm (excited at 315 nm). A related zinc acetate complex bearing 4′-phenyl-2,2′:6′,2″-terpyridine instead of the macrocyclic ligand displays two bands in its photoluminescent spectrum at 381 (high intensity with a high peak in the same spectrum) and 500 nm (low intensity, a low peak) [39]. The band in **3** can also be assigned as an ILCT according to the results in both compounds.

One band is clearly detected at 390 nm with a high intensity for benzoate complex **4**. In comparison with the zinc acetate 4′-phenyl-2,2′:6′,2″-terpyridine complex mentioned above, the peak, at the higher energy, is very close with similar intensity, being assigned to an ILCT band.

The benzoate compounds **5**, **6** and **7** with hydroxyl substituents also show interesting photoluminescent properties and the intensities of their bands and positions depending on the substituent of the hydroxyl group at the benzoate ligands. In the spectrum of **5**, excited at 355 nm, a band is observed at 425 nm, whereas a strong peak at 388 nm is displayed in the spectrum of **6**, excited at 304 nm. When excited at 300 nm, compound **7** shows its emission band at 411 nm. For complexes **4**–**7**, the bands are also assigned to an ILCT.

For the summarization of the results, these compounds present some regularities. For complexes **1** and **2**, because the sulfonate auxiliary ligands are electron withdrawing groups, the photoluminescent bands appear at the lowest energy and the emission bands give a great redshift compared to that of the ligand. As for carboxylate compounds (except for **5**), the photoluminescent bands appear at the higher energy than that of the ligand since the substituded carboxylate auxiliary ligands are electron donating groups, while the band of **5** is at the lower energy region than that of the ligand. All these carboxylate compounds give higher energy bands than those of the sulfonate compounds.

For complex **3**, on account of the influence of acetate auxiliary ligands, the methyl group at acetate has a negative induction. Its peak appears with the highest energy and the emission bands show a largely blueshifted phenomenon compared to that of the ligand.

The emission bands of complexes **4**–**7** display a slight change in comparison with that of the ligand since the phenyl group at benzoate has a positive induction effect. With the *meta*-OH substituent, the emission band of **6** is close to that of **4**. As for **7**, with the hydroxyl substituent at the *para*-position, the emission band is nearly identical to that of the ligand. But with the hydroxyl substituent at the *ortho*-position, the emission band of **5** undergoes a redshift from 413 to 425 nm. The sequence for the wavelength of the emission bands of **5**–**7** in solid state at room temperature is **5** > **7** > **6**.

The bands of the photoluminescence of the complexes are variety because the difference of the second ligands even if the mechanism to be the same as ILCT. Nevertheless, the clear rationale for the influence of hydroxyl substituent at the phenyl ring on the photoluminescence also cannot be established, conceivably relying on resonance and inductive effects, hydrogen bonds, interaction with the neighbouring molecules or packing of the molecules in the solution and the solid state. The peaks of photoluminescence in such compounds do not also comply with the sequence of pK_a_ values of the conjugated acids of the anions.

The photoluminescent properties of the complexes were also studied in solution. On account of their solubility in DMSO and DMF, these were the selected solvents (Table 3). The seven complexes give a strong photoluminescence (in comparision with other metal complexes, such as silver, copper, cadium and nickel ion ones) in the range of 390–446 nm or 379–440 both in DMSO or in DMF with the Exmax in the range of 308–354 or 300–360 nm and they just show one emission band in solution. For compounds **1** and **2**, the wavelength increases from ca. 411 to 440 nm, but the emission bands for **3** and **4** are nearly equal from DMSO to DMF. Meanwhile, **5**, **6** and **7** display one band decreasing from DMF to DMSO, especially for **6**, which differs greatly with a value of 67 nm.

The emission bands of these compounds in solution and in solid state at room temperature are completely different. As for DMF, the emission bands of all complexes exhibit redshifted results in comparison with that of the ligand. Interestingly, with the *meta*-OH substituent, the emission band of complex **6** shows the maximum wavelength in DMF and the minimal wavelength in DMSO, and the emission band is nearly identical to that of the ligand in DMSO. As for the sulfonate complexes, compounds **1** and **2** always exhibit the similar emission bands in DMF, DMSO or in solid state at room temperature and the results indicate that the groups at the sulfonate have no effect on the photoluminescence of these compounds. The photoluminescent bands of L, **1**, **2** and **3** demonstrate redshifted consequences, while these of **4**, **5**, **6** and **7** give the blue-shifted phenomena from DMF to DMSO. The sequence for the wavelength of the emission bands of **5**–**7** in DMSO is **5** > **7** > **6**, with the same result in solid state, but contrary to that of these compounds in DMF being **6** > **7** > **5**.

From the results obtained in these studies, all peaks appearing at the emission bands of photoluminescence are tentatively assigned to ILCT. The results also show that the charge transfers of the ligand in these compounds are affected by the anionic auxiliary ligands and the solvents used in the tests.

The fluorescence quantum yields (Φ) of the L and compounds in DMF and DMSO were measured according to the comparative method and the data were listed in Table 4. The results show that all compounds exhibit low Φ values in comparison with that of the standard sample l-Tryptophan (Φ_st_ = 0.15). Interestingly, the Φ values of **5**–**7** display the same trend in DMF or DMSO. With the hydroxyl substituent at the *ortho*-, *meta*- and *para*-position, the Φ values of **5**–**7** undergoe a greatly reduction from 0.1057 to 0.0005 in DMF and 0.1227 to 0.0105 in DMSO. Compound **5** displays the highest Φ value and compound **7** shows the lowest Φ value in DMF or DMSO. Each compound exhibits a higher Φ value in DMSO than that in DMF.

## 3. Materials and Methods

### 3.1. Instrumentation and Reagents

^1^H-NMR spectra were run on Unity 300 or 400 spectrometers (Varian, Lisbon, Portugal and Nanning, Guangxi, China). Elemental analyses were determined with an Elementar EL III Elemental Analyser (Vario, Lisbon, Portugal). IR spectra were measured with a Spectrum 2000 (Perkin-Elmer, Nanning, Guangxi, China) or a Magna 750 FT-IR (Nicolet, Lisbon, Portugal) spectrophotometer, in KBr pellets. UV-vis absorption spectra were measured with a DU 800 spectrophotometer (Beckman Coulter, Nanning, Guangxi, China). Emission and excitation spectra were recorded on a Perkin-Elmer LS 55 luminescence spectrometer with a red-sensitive photomultiplier type R928. TG-DTA data were collected with a Perkin Elmer STA6000 Simultaneous Thermal Analyzer at a heating rate of 10 K min^−1^ under an air atmosphere. All reagents used in the experiments were of analytical grade or purified by standard methods.

### 3.2. Synthesis of L^0^

The precursor L^0^ was prepared by following a reported procedure [41]. Zn(SO_3_CF_3_)_2_ was a product of Fluka (Kenilworth, NJ, USA) and Zn(OCOCH_3_)_2_·2H_2_O was a product of Riedel-de Haën (Shanghai, China). Zn(OCOPh)_2_ was synthesized by the reaction of Zn(NO)_2_·6H_2_O and NaCOOPh in water. Other salts were prepared by the reaction of Zn(OH)_2_ and the respective acid.

### 3.3. Synthesis of L^1^

L^0^ (1.0 g, 1.5 mmol) and methanol (400 mL) were mixed in a 1000 mL flask and a white emulsion was obtained. KBH_4_ (2.0 g, 37 mmol) was then added in portions of 400 mg every 20 min. The colourless solution thus obtained was stirred for half an hour, then filtered and the solution taken to dryness. Upon addition of 200 mL of water it was left stirring for 1 h. The white solid was isolated by filtration, recrystallized from methanol and dried under vacuum (0.90 g, yield 89%), m.p. 177–179 °C. Anal. calcd for L^1^·CH_4_O·2.5H_2_O (C_41_H_63_N_6_O_7.5_) C, 64.80; H, 8.36; N, 11.06. Found C, 64.87; H, 8.47; N, 10.82; ^1^H-NMR (400 MHz, CD_3_OD:CDCl_3_ = 1:4), *δ* 2.55 (s, 16H, NH–CH_2_), 3.50 (s, 8H, CH_2_–Ph), 4.09 (s, 8H, O–CH_2_), 6.69 (d, *J* = 8.4 Hz, 8H, aryl), 7.00 (d, *J* = 8.4 Hz, 8H, aryl). ^13^C-NMR (100 MHz, CD_3_OD:CDCl_3_ = 1:4): *δ* 47.59 (NH–CH_2_CH_2_), 47.68 (NH-CH_2_CH_2_), 52.59 (CH_2_–Ph), 66.41 (O–CH_2_), 114.32 (aryl–O), 129.24 (aryl), 131.86 (aryl), 157.61 (aryl–CH_2_). IR (KBr disc) (cm^−1^): 2932 (m), 2831 (m), 1611 (s, υ_aryl-H_), 1515 (vs, υ_aryl-H_), 1453 (s), 1380 (w), 1299 (m), 1243 (vs), 1179 (m), 1113 (w), 1068 (s), 939 (s), 810 (s), 770 (m), 616 (w), 522 (w).

### 3.4. General Synthetic Procedure for the Preparation of Zinc Compounds (Scheme 1)

Compounds [Zn_2_L^1^ (DMSO)_4_](OSO_2_CF_3_)_4_ (**1**), [Zn_2_(*p*-OSO_2_PhCH_3_)_4_L^1^] (**2**), [Zn_2_(OCOCH_3_)_4_L^1^] (**3**), [Zn_2_(OCOPh)_4_L^1^] (**4**), [Zn_2_(*o*-OCOPhOH)_4_L^1^] (**5**), [Zn_2_(*m*-OCOPhOH)_4_ L^1^] (**6**) and [Zn_2_(*p*-OCOPhOH)_4_L^1^] (**7**), were synthesized by reaction of L^1^ with the appropriate Zn(II) salt added in stoichiometric amounts, i.e., Zn(SO_3_CF_3_)_2_, Zn(*p*-SO_3_PhCH_3_)_2_·6H_2_O, Zn(OCOCH_3_)_2_, Zn(OCOPh)_2_, Zn(*o*-OCOPhOH)_2_·2H_2_O, Zn(*m*-OCOPhOH)_2_·2H_2_O or Zn(*p*-OCOPhOH)_2_·3MeOH·1.5H_2_O, respectively, in a methanol—CH_2_Cl_2_ solution (**1**, **2** (method 2), **3**, **4**), DMF (**2**, method 1) or a DMF—methanol—CH_2_Cl_2_ solution (**5**, **6**, **7**). The reactions usually proceeded smoothly at room temperature and the complexes were isolated after 24 h, in good to high yields (56–88%).

#### 3.4.1. [Zn_2_L^1^ (DMSO)_4_](OSO_2_CF_3_)_4_ (**1**)

A white solid (0.56 g, 88% based on the ligand) was formed by adding diethyl ether to the filtrate. The solid was dissolved in DMSO at 80 °C. Cooling the system gave the colorless crystals, which were suitable for X-ray analysis (0.23 g, 36% based on the ligand). Anal. calcd for C_56_H_90_F_12_N_6_O_22_S_10_Zn_2_: C 35.80; H 4.83; N 4.47. Found: C 35.67; H 4.76; N 4.31. TG-DTA determination: DMSO % = 24.0, ZnO % = 11.6; Calc by formula C_42_H_54_F_12_N_6_O_16_S_4_Zn_2_·C_12_H_36_S_6_O_6_: DMSO % = 25.0, ZnO % = 8.7. ^1^H-NMR (300 MHz, DMSO-*d*_6_): *δ* 2.27 (s, 4H) 2.78 (s, 4H), 3.50–3.64 (m, 16H), 4.28 (s, 8H), 6.97 (d, *J* = 8.2 Hz, 8H), 7.23 (d, *J* = 7.8 Hz, 8H). ^13^C-NMR (75 MHz, DMSO-*d*_6_): *δ* 44.28, 50.95, 66.50, 114.37, 114.66, 118.64, 122.91, 127.18, 129.09, 131.11, 158.04. IR (KBr disc) (cm^−1^): 3251 (m), 3014 (m), 2929, 2879, 1612 (s, υ_aryl-H_), 1515 (s, υ_aryl-H_), 1454 (m), 1384 (s, υ_aryl-H_), 1274 (vs), 1157 (s), 1031 (vs), 953 (s), 847 (m), 819 (m), 763 (m), 707 (w), 639 (vs), 573 (m), 517 (s), 472 (w), 456 (m), 439 (m), 417 (m), 407 (m). λ_max_ (DMF)/nm 274 (*ε*/M^−1^·cm^−1^, 4816), λ_max_ (DMSO)/nm 273 (*ε*/M^−1^·cm^−1^, 4100).

#### 3.4.2. [Zn_2_(*p*-OSO_2_PhCH_3_)_4_L^1^] (**2**)

Method 1: Diffusion of a colorless solution formed with a mixture of diethyl ether and 1,4-dioxane led to the formation of colorless crystals (0.31 g, 51% based the ligand). Anal. calcd for C_68_H_82_N_6_O_16_S_4_Zn_2_·3DMF·H_2_O: C 53.28, H 6.10, N 7.26. Found: C 53.14; H 6.38; N 7.43. TG-DTA: ZnO % = 11.4, DMF % = 15.9; Calc by formula C_68_H_82_N_6_O_16_S_4_Zn_2_·4DMF: ZnO % = 9.1, DMF % = 16.3. ^1^H-NMR (300 MHz, DMSO-*d*_6_): *δ* 2.30 (s, 16H), 2.41 (s, 4H), 2.72 (s, 12H, CH_3_-DMF), 2.88 (s, 16H, C*H_3_-DMF*), 3.56 (s, 4H), 3.76 (s, 8H), 3.87 (s, 4H), 4.32 (s, 8H), 6.93 (s, 8H), 7.16 (d, *J* = 5.9 Hz, 8H), 7.24 (d, *J* = 5.6 Hz, 8H), 7.53 (d, *J* = 5.9 Hz, 8H), 7.94 (s, 4H, COH-DMF). ^13^C-NMR (75 MHz, DMSO-*d*_6_): *δ* 20.82, 30.78, 35.80, 44.08, 44.43, 50.84, 66.37, 114.74, 114.90, 125.54, 128.30, 129.34, 130.71, 138.32, 144.73, 157.79, 162.35. IR (KBr disc) (cm^−1^): 3303 (w), 3246 (s), 3069 (w), 3022 (w), 2925 (vs), 2880 (s), 1656 (vs, υ_DMF=O_), 1611 (s, υ_aryl-H_), 1513 (s, υ_aryl-H_), 1453 (s), 1385 (s, υ_aryl-H_), 1245 (s), 1218 (s), 1183 (s), 1120 (s), 1071 (w), 1034 (s), 1010 (s), 959 (m), 917 (w), 818 (vs), 771 (w), 735 (w), 733 (w), 683 (vs), 614 (m), 566 (s). λ_max_ (DMF)/nm 272 (*ε*/M^−1^·cm^−1^, 2636), λ_max_ (DMSO)/nm 273 (*ε*/M^−1^·cm^−1^, 3524).

Method 2: Concentration and diffusion of a colorless solution formed with diethyl ether led to the formation of white solid. (0.53 g, 80% based the ligand). ^1^H-NMR (400 MHz, DMSO-*d*_6_): *δ* 2.29 (s, 16H), 2.40 (s, 4H), 2.84 (s, 4H), 3.58 (s, 4H), 3.77 & 3.84 (s, 8H), 4.32 (s, 8H), 6.92 (s, 8H), 7.16 (d, *J* = 7.7 Hz, 8H), 7.24 (d, *J* = 7.6 Hz, 8H), 7.54 (d, *J* = 7.8 Hz, 8H). ^13^C-NMR (75 MHz, DMSO-*d*_6_): *δ* 20.88, 44.12, 44.46, 50.87, 66.43, 66.54, 114.82, 115.01, 125.61, 128.41, 129.43, 130.74, 138.52, 144.57, 157.82.

#### 3.4.3. [Zn_2_(OCOCH_3_)_4_L^1^] (**3**)

A white solid (0.15 g, 86% based on the ligand) was formed by adding diethyl ether to the filtrate. Calc by formula C_48_H_66_N_6_O_12_Zn_2_·CH_4_O·CH_2_Cl_2_·1.2H_2_O: C 50.53, H 6.31, N 7.07. Found: C 50.61, H 6.28, N 6.97. The slow diffusion of diethyl ether into the solution of the complex (0.20 g) in DMF led to the formation, after several days, of colourless crystals which were suitable for X-ray analyses (0.10 g, yield 50% based on L^1^). TG-DTA: ZnO % = 12.4%, Weight % (loss of H_2_O and DMF) = 16.2. Calc with the formula of C_48_H_66_N_6_O_12_Zn_2_·2C_3_H_7_NO·4H_2_O: ZnO % = 12.8, Weight % (loss of H_2_O and DMF) = 17.2. ^1^H-NMR (400 MHz, DMSO-*d*_6_): *δ* 1.75 (s, 12H, CO_2_CH_3_), 2.53 (s, 8H, CH_2_–N), 2.85 (s, 10H, CH_3_–DMF & CH_2_–N), 2.98 (s, 10H, CH_3_–DMF & CH_2_–N), 3.56 & 3.75 (d, 8H, Ar–CH_2_–N), 4.34 (s, 8H, Ar–CH_2_–O), 6.91 (d, 8H, *J* = 8.5 Hz, –Ar), 7.20 (d, 8H, *J* = 8.5 Hz, –Ar), 7.95 (s, 2H, CHO–DMF). ^13^C-NMR (75 MHz, DMSO-*d*_6_): *δ* 23.22 (CO_2_*C*H_3_), 31.65 (CH_3_–DMF), 36.95 (CH_3_–DMF), 46.79, 47.46, 53.15, 67.55, 66.54, 116.29, 131.41, 131.49, 159.36, 164.84 (CHO–DMF), 180.68 (CO_2_CH_3_). Or slow evaporation of the solution of the complex (0.20 g) in the mixture of methanol and 1,4-dioxane led to the formation, after several days, of colourless crystals (0.16 g, 80%). Calc by formula C_48_H_66_N_6_O_12_Zn_2_·C_4_H_8_O_2_·2.5H_2_O C 52.79, H 6.73, N 7.10. Found C 52.90, H 6.58, N 7.05. IR (KBr disc) (cm^−1^): 3257 (s), 2934 (s), 2876 (s), 1612 (vs, υ_aryl-H_), 1562 (vs), 1512 (vs, υ_aryl-H_), 1415 (vs), 1333 (m, υ_aryl-H_), 1305 (m), 1247 (vs), 1219 (s), 1177 (s), 1115 (w), 1097 (w), 1075 (s), 1015 (m), 983 (w), 949 (m), 921 (m), 817 (s), 761 (w), 667 (s), 619 (m), 568 (w), 550 (w), 532 (w). λ_max_ (DMF)/nm 274 (*ε*/M^−1^·cm^−1^, 4159), λ_max_ (DMSO)/nm 273 (*ε*/M^−1^·cm^−1^, 2603).

#### 3.4.4. [Zn_2_(OCOPh)_4_L^1^] (**4**)

Evaporation naturally of the filtrate gives colorless crystals (0.17g, 56% based the ligand). Anal. calcd for C_68_H_74_N_6_O_12_Zn_2_·3.5H_2_O: C 60.00, H 6.00, N 6.17. Found: C 59.96; H 6.02; N 5.96. TG-DTA: ZnO % = 12.5%, Weight % (loss of H_2_O) = 5.1. Calc with the formula of C_68_H_74_N_6_Zn_2_O_16_·3.5H_2_O: ZnO % = 12.0, Weight % (loss of H_2_O) = 4.6. IR (KBr disc) (cm^−1^): 3272 (m), 3064 (w), 2926 (s), 2871 (m), 1604 (vs, υ_aryl-H_), 1557 (s), 1514 (s, υ_aryl-H_), 1455 (m), 1385 (vs, υ_aryl-H_), 1303 (w), 1245 (vs), 1180 (m), 1117 (w), 1069 (s), 1023 (m), 943 (s), 821 (s), 771 (w), 723 (vs), 680 (m), 617 (w), 564 (m), 516 (w), 429 (m). λ_max_ (DMF)/nm 272 (*ε*/M^−1^·cm^−1^, 818), λ_max_ (DMSO)/nm 271 (*ε*/M^−1^·cm^−1^, 1269).

#### 3.4.5. [Zn_2_(*o*-OCOPhOH)_4_L^1^] (**5**)

A white solid (0.49 g, 82% based on the ligand) was formed by filtering the reaction mixture. Anal. calcd for C_68_H_74_N_6_Zn_2_O_16_·2C_3_H_7_NO·0.5H_2_O: C 58.57, H 5.91, N 7.38. Found C 58.40, H 5.95, N 7.40. TG-DTA: Determined ZnO % = 10.6, Solvents % = 9.2; Calc for C_68_H_74_N_6_Zn_2_O_16_·2C_3_H_7_NO·H_2_O: ZnO % = 10.7 Solvents % = 10.2. The solid obtained was dissolved in 20 mL DMF at 80 °C and cooling this solution to room temperature led to the formation of colorless crystals (0.18 g with yield 37%). ^1^H-NMR (400 MHz, DMSO-*d*_6_): *δ* 2.73 (s, 12H), 2.89 (s, 16H), 3.01 (s, 4H), 3.57 (s, 8H), 4.21 (s, 8H), 6.75 (m, 8H), 6.83 (d, 8H, *J* = 8.4 Hz), 7.30 (m, 12H), 7.61 & 7.62 (s, 8H), 7.65 (s, 4H), 13.48 (s, 4H, Ar–OH). ^13^C-NMR (100 MHz, DMSO-*d*_6_): *δ* 30.78 (CH_3_–DMF), 35.79 (CH_3_–DMF), 44.12, 44.46, 50.87, 66.23, 114.10, 116.04, 117.40, 129.57, 130.64, 130.74, 132.91, 157.68, 161.41, 162.32 (COH-DMF), 173.18 (CO_2_–Ar). IR (KBr disc) (cm^−1^): 3262 (m), 2927 (s), 2863 (s), 1627 (vs, υ_aryl-H_), 1568 (m), 1514 (s, υ_aryl-H_), 1486 (s), 1455 (s), 1391 (vs, υ_aryl-H_), 1338 (w), 1307 (m), 1247 (s), 1180 (m), 1117 (w), 1070 (s), 1030 (m), 942 (m), 862 (m), 811 (m), 763 (m), 705 (m), 670 (m), 615 (m), 561 (w), 536 (w), 420 (w). λ_max_ (DMF)/nm 294 (*ε*/M^−1^·cm^−1^, 4091), λ_max_ (DMSO)/nm 291 (*ε*/M^−1^·cm^−1^, 3709).

#### 3.4.6. [Zn_2_(*m*-OCOPhOH)_4_L^1^] (**6**)

Filtration led to the separation of a white powder of **6** from its mother solution (0.52 g, 87%). Anal. calcd for C_68_H_74_N_6_Zn_2_O_16_·2C_3_H_7_NO·CH_2_Cl_2_·H_2_O: C 55.91; H 5.76; N 6.95. Found: C 55.86; H 5.70; N 7.10. The solid was dissolved in DMSO at 80 °C and colorless crystals were obtained by cooling of the system to room temperature (0.27 g, 45%). TG-DTA: Determined ZnO % = 10.9, Solvents % = 8.8; Calc by formula C_68_H_74_N_6_Zn_2_O_16_·C_4_H_12_S_2_O_2_: ZnO % = 10.7, Solvents % = 10.3. ^1^H-NMR (400 MHz, DMSO-*d*_6_): *δ* 2.75 (m, 16H), 3.53 (s, 8H), 4.17 (s, 8H), 6.77 (m, 12H), 7.06 (m, 8H), 7.31 (m, 12H), 9.32 (s, 4H, Ar–OH). ^13^C-NMR (100 MHz, DMSO-*d*_6_): *δ* 46.21, 48.12, 52.21, 66.26, 113.63, 116.26, 117.08, 120.37, 128.22, 130.11, 130.58, 137.86, 156.69, 157.48, 170.81 (CO_2_–Ar). IR (KBr disc) (cm^−1^): 2943 (m), 2872 (w), 1618 (s, υ_aryl-H_), 1554 (s), 1514 (s, υ_aryl-H_), 1446 (m), 1391 (vs, υ_aryl-H_), 1303 (m), 1231 (vs), 1186 (m), 1120 (w), 1072 (s), 1014 (s), 980 (w), 946 (m), 798 (m), 773 (s), 672 (w), 566 (w). λ_max_ (DMF)/nm 282 (*ε*/M^−1^·cm^−1^, 5185), λ_max_ (DMSO)/nm 283 (*ε*/M^−1^·cm^−1^, 3454).

#### 3.4.7. [Zn_2_(*p*-OCOPhOH)_4_L^1^] (**7**)

Filtration led to the separation of a white powder of **7** from its mother solution (0.51 g, 85%). Anal. calcd for C_68_H_74_N_6_O_16_Zn_2_·4C_3_H_7_NO·0.5CH_2_Cl_2_·0.5H_2_O (in DMF): C 56.67; H 6.14; N 8.21. Found: C 56.74; H 6.17; N 8.02. TG-DTA: Determined: solvents % = 13.2, ZnO % = 11.0; Calc by formula C_68_H_74_N_6_O_16_Zn_2_·0.5C_2_H_6_SO·1.5C_3_H_7_NO·0.5CH_2_Cl_2_·0.5H_2_O (in DMF + DMSO): Solvents % = 12.8, ZnO % = 10.4. 0.20 g of the solid was dissolved in 10 mL DMF and 5 mL H_2_O at 80 °C. Cooling of the system led to the formation of colorless crystals, which were suitable for X-ray analysis. ^1^H-NMR (400 MHz, DMSO-*d*_6_): *δ* 2.73 (s, 18 H), 2.88 (s, 16H), 3.54 (s, 8H), 4.20 (s, 8H), 6.72 (d, 8H, *J* = 8.0 Hz), 6.82 (d, 8H, *J* = 8.4 Hz), 7.39 (d, 8H, *J* = 7.4 Hz), 7.60 (d, 8H), 7.95 (s, 3H), 9.77 (s, 4H, Ar-OH). ^13^C-NMR (100 MHz, DMSO-*d*_6_): *δ* 30.78 (CH_3_–DMF), 35.79 (CH_3_–DMF), 46.45, 47.60, 52.36, 66.31, 113.82, 114.04, 127.26, 130.15, 130.82, 131.31, 157.55, 162.33 (COH–DMF), 170.89 (CO_2_-Ar). IR (KBr disc) (cm^−1^): 2943 (m), 2858 (w), 1603 (s, υ_aryl-H_), 1558 (s), 1513 (s, υ_aryl-H_), 1455 (m), 1382 (vs, υ_aryl-H_), 1275 (s), 1247 (s), 1182 (m), 1165 (m), 1069 (s), 1015 (m), 952 (m), 858 (m), 833 (m), 798 (m), 788 (s), 704 (w), 635 (m), 563 (w), 512 (w). λ_max_ (DMF)/nm 277 (*ε*/M^−1^·cm^−1^, 4111), λ_max_ (DMSO)/nm 274 (*ε*/M^−1^·cm^−1^, 3167).

### 3.5. Crystal Structure Determinations

Single crystals of **1**–**7** were obtained as indicated above. Intensity data were collected using a Bruker AXS-KAPPA APEX II (Nanning, Guangxi, China and Lisbon, Portugal) or Agilent SuperNova (Dual, Atlas, Nanning, Guangxi, China) diffractometer with graphite monochromated Mo-Kα (λ = 0.71073 Å) radiation. Data were collected using omega scans of 0.5° per frame and full sphere of data were obtained. For the former, cell parameters were retrieved using Bruker SMART software and refined using Bruker SAINT [42] on all the observed reflections. Absorption corrections were applied using SADABS [42]. For the latter, cell parameters were retrieved using Agilent CrysAlisPro [43] software and refined using Agilent CrysAlisPro [43] on all the observed reflections. Structures were solved by direct methods by using the SHELXS-97 package [44] and refined with SHELXL-97 [45]. Calculations were performed using the WinGX System-Version 1.80.03 [46]. All hydrogens were inserted in calculated positions. Least square refinements with anisotropic thermal motion parameters for all the non-hydrogen atoms and isotropic for the remaining atoms were employed. CCDC 846596, 968278, 968279, 968280, 968281, 968282 and 1526883 contain the supplementary crystallographic data for this paper. These data can be obtained free of charge from the Cambridge Crystallographic Data Centre via www.ccdc.cam.ac.uk/data_request/cif.

### 3.6. Photoluminescent Property Measurement

Emission and excitation spectra were recorded on a Perkin–Elmer LS 55 luminescence spectrometer with a red-sensitive photomultiplier type R928. The amounts of the solid state samples used in the photoluminescent studies were as follows: 5 mg for compounds **1** and **2**, and 20 mg for compounds **3**–**7**. The scan rate is 50 nm/s. The solutions of the compounds were prepared in 10 mL volumetric flask. The concentration of the compounds (L^1^, **1**–**3**, **5**, **7**) is 1 mg/mL in DMF or DMSO, which is 0.5 mg/mL for **4** and **6**. When the solutions were prepared, the solids of **4** and **6** were dissolved in DMF or DMSO and heat a little beat until they are soluble in these solvents. The measurement of solution is carried out in 1 cm cuvette. The conditions for the solution tests are equal to these of solid samples.

The fluorescence quantum yields were measured according to the comparative method of Williams et al. [47], and the l-Tryptophan was chosen as a standard sample. The Φ values of samples were calculated according to the following equation:

$$\Phi_{x}=\Phi_{\mathrm{st}}\times \left(\frac{{\mathrm{Grad}}_{x}}{{\mathrm{Grad}}_{\mathrm{st}}}\right)\times \left(\frac{\eta_{x}^{2}}{\eta_{\mathrm{st}}^{2}}\right)$$

where the subscripts st and x denote standard and test samples respectively, Φ is the fluorescence quantum yield, Grad is the gradient from the plot of integrated fluorescence intensity vs. absorbance, and η is the refractive index of the solvent. The UV-vis absorbance spectra and fluorescence spectra of l-Tryptophan (in ultrapure water) and compounds (in DMF and DMSO) in different concentrations were recorded in the same test conditions. The wavelength of 270 nm was chosen for determination of absorbance and integrated fluorescence intensity and the data of the results were used for calculation. Φ_st_ is got from the liteature [48].

## 4. Conclusions

The N_6_O_4_ macrocyclic ligand is indicated as a convenient *N*,*N*,*N*,*N*,*N*,*N*-hexapodal ligand source for the facile synthesis of Zn (II) complexes with interesting structures. Seven Zn(II) complexes with this ligand were synthesized (usually in good yields) by simple reactions with the respective zinc salts, and in their formulae, the auxiliary anionic ligand was decided by the nature of the salt.

The prepared zinc complexes show photoluminescence in the solid state and in DMSO or DMF at room temperature and the photoluminescent properties (i.e., the energy and intensity of the emission peaks) are distinctive in accordance with the variation of the anionic auxiliary ligands and the solvents used in the determination. The emission bands of sulfonate complexes appear at the lowest energy and show greatly red-shifted behaviours in comparison with that of the ligand, while the peaks of carboxylate compounds appear at the higher energy than that of the ligand since the electron withdrawing capability of the carboxylate auxiliary ligand is weaker than that of sulfonate. The influence of the hydroxyl substituent on the photoluminesce of benzoate compounds greatly relies on the position of hydroxyl on the aryl ring. All these carboxylate compounds show higher energy than the sulfonate compounds. The groups at the sulfonate have no effect on the photoluminescence of these compounds. The determination of fluorescence quantum yields shows that all compounds exhibit low Φ values in comparison with that of the standard sample. The results show that the countions as auxiliary ligands have great influences on the photoluminescent properties of the compounds.

The thermal properties of the compounds are also highly affected by the second ligands. The sulfonate complexes show a medium final stable temperature in comparison with the carboxylate ones. Within the latter, the *meta*-position of the hydroxyl group (compound **6**) results in the lowest thermal stability.

However, the results of the above types of effects can be of importance towards further design of photoluminescent materials. The work provides good roles for further photoluminescent and thermal studies and deserves to be extended to other macrocyclic zinc complexes on attempting to study substituent effects and to establish relationships.

## Figures and Tables

**Table 1:** Crystallographic data for compounds **1**–**7**.

	1·2DMSO	2·4DMF·C_4_H_8_O_2_·H_2_O	3·2DMF·4H_2_O	4·2CH_4_O·3H_2_O	5·2CH_4_O·2H_2_O	6·4DMSO	7·2H_2_O
Formula	C_56_H_90_F_12_N_6_O_22_S_10_ Zn_2_	C_88.5_H_123.5_N_11.5_O_24_S_4_Zn_2_	C_54_H_86_N_8_O_18_Zn_2_	C_140_H_170_N_12_O_31_Zn_4_	C_70_H_84_N_6_O_19_Zn_2_	C_76_H_98_N_6_O_20_S_4_Zn_2_	C_68_H_78_N_6_O_18_Zn_2_
Formula Weight	1878.82	1991.55	1266.09	2778.36	1444.21	1674.62	1398.10
Crystal system	Monoclinic	Triclinic	Monoclinic	Monoclinic	Triclinic	Triclinic	Triclinic
Crystal size (mm)	0.39 × 0.37 × 0.31	0.45 × 0.39 × 0.32	0.46 × 0.41 × 0.29	0.39 × 0.26 × 0.22	0.43 × 0.40 × 0.32	0.46 × 0.38 × 0.35	0.39 × 0.36 × 0.28
Space group	*P*21/*c*	*P*-1	*P*21/*c*	*P*2(1)/*c*	*P*-1	*P*-1	*P*-1
*a* (Å)	12.5300(12)	14.7692(3)	9.6799(4)	18.596(4)	11.0068(4)	11.6847(5)	10.2345(4)
*b* (Å)	20.941(2)	18.9886(4)	16.4617(8)	9.6191(19)	11.8259(4)	11.7317(5)	15.3764(8)
*c* (Å)	18.1657(16)	19.1344(4)	20.8877(9)	39.263(8)	16.1201(5)	15.8174(6)	22.3239(12)
*α* (°)	90	90.3180(10)	90	90	74.221(3)	90.703(2)	86.275(4)
*β* (°)	123.313(5)	107.6140(10)	110.758(2)	91.81(3)	87.726(3)	93.460(2)	89.799(4)
*γ* (°)	90	104.8680(10)	90	90	64.877(3)	114.094(2)	73.514(4)
*V* (Å^3^)	3983.3(6)	4923.15(18)	3112.3(2)	7020(2)	1821.26(11)	1974.13(14)	3361.2(3)
*Z*	2	2	2	2	1	1	2
*D_calc_* (Mg m^−3^)	1.566	1.343	1.351	1.314	1.317	1.409	1.381
*μ* (mm^−1^)	0.964	0.648	0.844	0.752	0.731	0.788	0.789
*F* (000)	1944	2120	1340	2924	758	880	1464
No. *R*fl. unique	12158	29649	8393	17545	9039	8948	16660
No. *R*fl. observed	10353	21994	6634	9294	7033	5891	8622
*R*	0.0502	0.0550	0.0365	0.0607	0.0615	0.0440	0.0709
*wR*	0.1388	0.1615	0.1152	0.1403	0.1871	0.1063	0.1504
*R for all*	0.0596	0.0794	0.0520	0.1282	0.0792	0.0952	0.1487
*wR for all*	0.1457	0.1827	0.1258	0.1756	0.2047	0.1397	0.1934
GOF	1.019	1.008	1.022	1.005	1.014	1.018	1.013
CCDC number	968282	1526883	846596	968278	968279	968280	968281

**Table 2:** Decomposition processes in TG-DTA of the benzoate complexes **4**–**7**.

Complex		4	5	6	7
Step 1	a, °C	60–126	120–200	130–158	60–215
	b, %	5.1	9.2	8.8	13.5
Step 2	a, °C	276–422	249–454	256–463	236–448
	b, %	44.2	45.6	30.2	38.3
Step 3	a, °C	422–674	454–550	463–518	448–569
	b, %	38.2	31.5	14.4	36.0
Step 4	a, °C			518–593	
	b, %			24.2	

a Temperature range of decomposition in TG-DTA of the complex; b Percentage of weight loss in every decomposition process of the complex.

**Table 3:** Photoluminescent properties of the compounds in solid state and solution.

		Bands, nm	L^1^	1	2	3	4	5	6	7
Solid state		Exmax	310	340	360	315	350	355	304	300
		Emmax	413	463	462	383	390	425	388	411
Solvent	DMF ^a^	Exmax	306	342	310	308	309	344	354	327
		Emmax	356	411	412	390	399	416	446	425
	DMSO ^a^	Exmax	300	350	360	300	300	325	315	330
		Emmax	380	440	440	395	396	408	379	402

^a^ The concentration of **4** and **6** are 0.5 mg/mL. When prepared, the solids of the two compounds were dissolved in DMF and DMSO and heat a little beat until they are soluble in these solvents. The concentrations of the other complexes prepared in the tests are 1 mg/mL in DMF and DMSO.

**Table 4:** The fluorescence quantum yields of the complounds in solution ^a^.

Solvent	Parameters	L^1^	1	2	3	4	5	6	7
DMF	Grad_x_	30,664	212,755	221,420	233,883	40,170	766,539	38,980	3542
	R^2^	0.9991	0.9989	0.9919	0.9994	0.9990	0.9989	0.9883	0.9952
	Φ	0.0042	0.0293	0.0305	0.0322	0.0055	0.1057	0.0054	0.0005
DMSO	Grad	138,805	319,677	281,248	95,396	265,597	829,585	126,016	71,006
	R^2^	0.9986	0.9900	0.9996	0.9916	0.9972	0.9977	0.9920	0.9939
	Φ_x_	0.0205	0.0473	0.0416	0.0141	0.0393	0.1227	0.0186	0.0105

^a^ Grad_x_ is the gradient of the compounds from the plot of integrated fluorescence intensity vs absorbance, R^2^ is the root-mean-square, Φx is the fluorescence quantum yield of the compounds. The Grad and R^2^ values of l-Tryptophan in ultrapure water are 1,249,037 and 0.9992.

## Supplementary Materials

Supplementary materials are available online. Figure S1: The TG-DTA curves of compound 3; Figure S2: The TG-DTA curves of compound 4; Figure S3: The TG-DTA curves of compound 5; Figure S4: The TG-DTA curves of compound 6; Figure S5: The TG-DTA curves of compound 7; Figure S6: Photoluminescent properties of the L^1^ and complexes **1**–**7** in DMF; Figure S7: Photoluminescent properties of the L^1^ and complexes **1**–**7** in DMSO; Figure S8: The UV-vis absorbance spectrum of the L^1^ and complexes **1**–**7** in DMF; Figure S9: The UV-vis absorbance spectrum of the L^1^ and complexes **1**–**7** in DMSO.
