# Supplementary material for: Study on the Photoluminescent and Thermal Properties of Zinc Complexes with a N6O4 Macrocyclic Ligand
Source: Molecules. 2018 Jul 16;23(7):1735. doi: 10.3390/molecules23071735 (PMC6100202; doi:10.3390/molecules23071735)
Supplement: Supplementary file 1 [file molecules-23-01735-s001.pdf]

Supplementary Materials

Article

Study on Photoluminescent and Thermal Properties of Zinc Complexes with a N6O4 Macrocyclic Ligand

Xingyong Xue [1,2], Qijun Wang [1], Fusen Mai [1], Xing Liang [1], Yichen Huang [1], Jiahe Li [1], Yanling Zhou [1], Dengfeng Yang [3,*], Zhen Ma [1,4,*]

[1] *School of Chemistry and Chemical Engineering, Guangxi University, Nanning 530004, P. R. China, E-mail: mzmz2009@sohu.com*
[2] *Guangxi Colleges and Universities Key Laboratory of Applied Chemistry Technology and Resource Development*
[3] *Guangxi Academy of Sciences, Guangxi beibu gulf marine research center, Guangxi key laboratory of marine natural products and combinatorial biosynthesis chemistry, Nanning, Guangxi, P. R. China, E-mail: yangdengfeng@gxas.cn*
[4] *Centro de Química Estrutural, Complexo I, Instituto Superior Técnico, Universidade de Lisboa, Av. Rovisco Pais, 1049-001, Lisbon, Portugal*
* Correspondence: *mzmz2009@sohu.com*; Tel.: +86-0771-323-3718

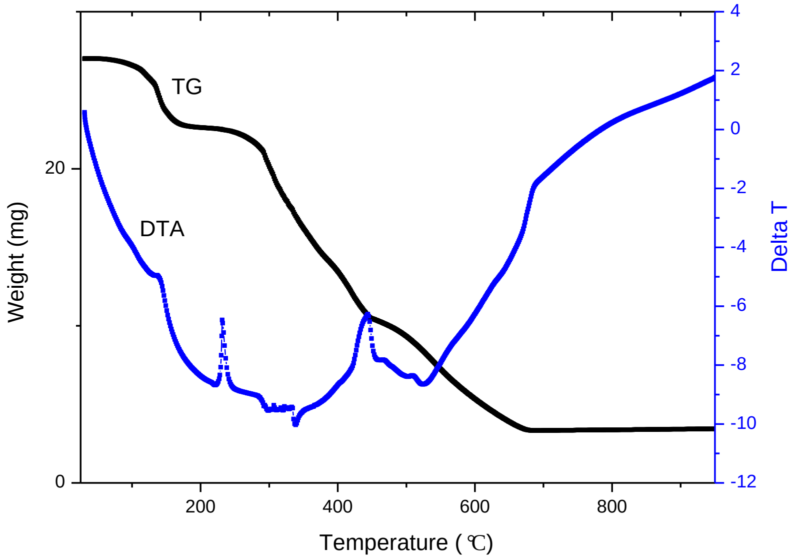

Figure S1. The TG-DTA curves of compound **3**

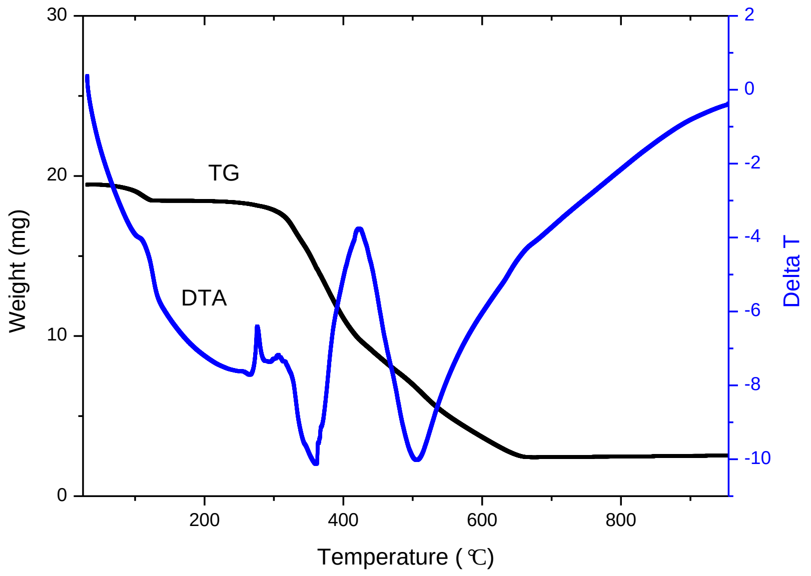

Figure S2. The TG-DTA curves of compound **4**

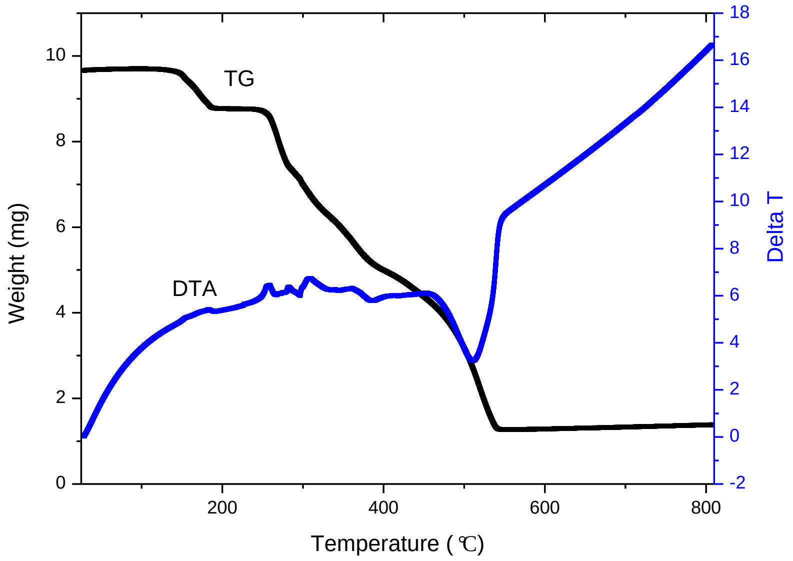

Figure S3. The TG-DTA curves of compound **5**

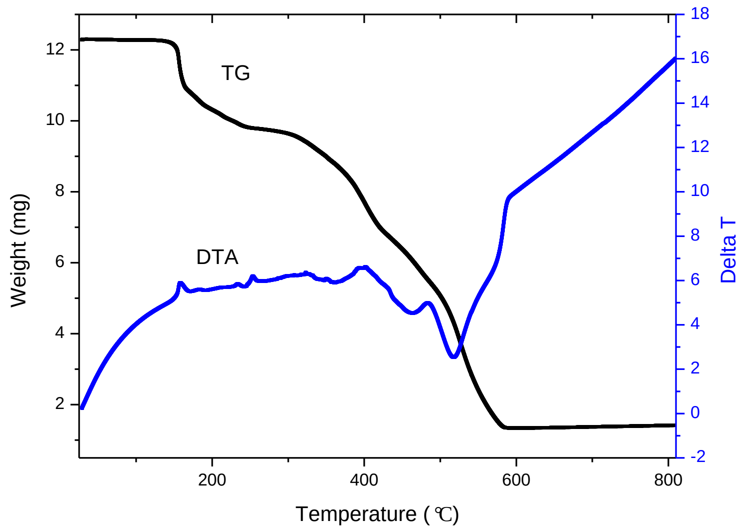

Figure S4. The TG-DTA curves of compound **6**

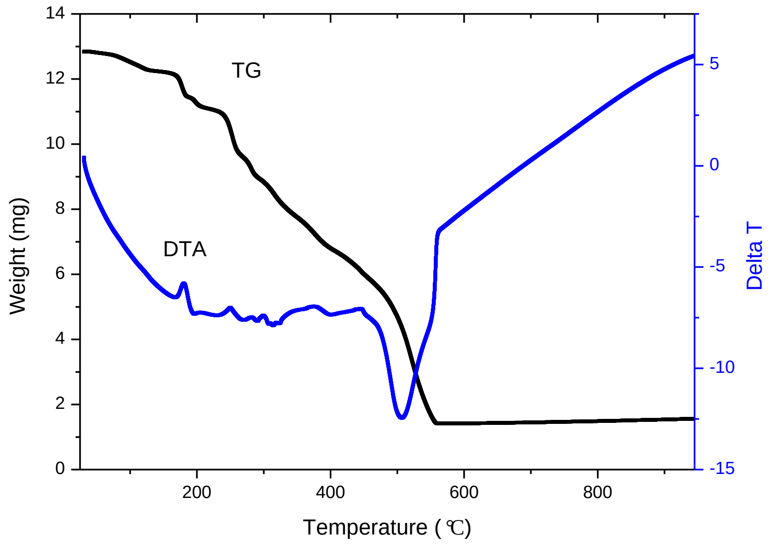

Figure S5. The TG-DTA curves of compound **7**

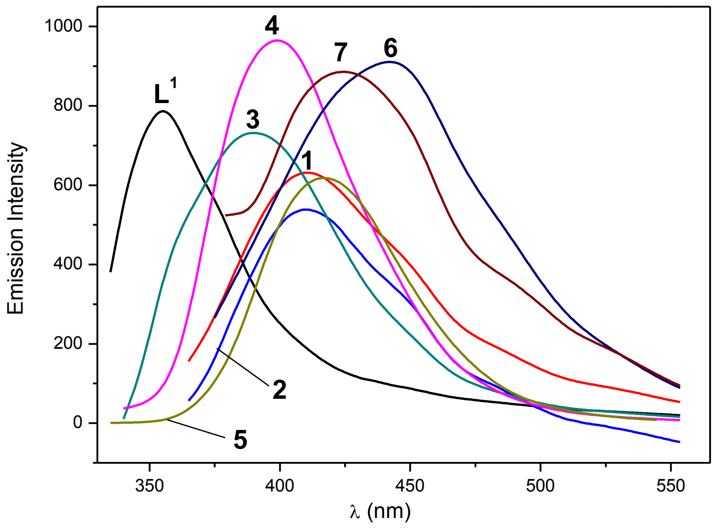

Figure S6. Photoluminescent properties of the L[1] and complexes **1-7** in DMF. The peak of emission is 356 nm for L[1], 411 (**1**), 412 (**2**), 390 (**3**), 399 (**4**), 416(**5**), 446(**6**), 425(**7**), respectively.

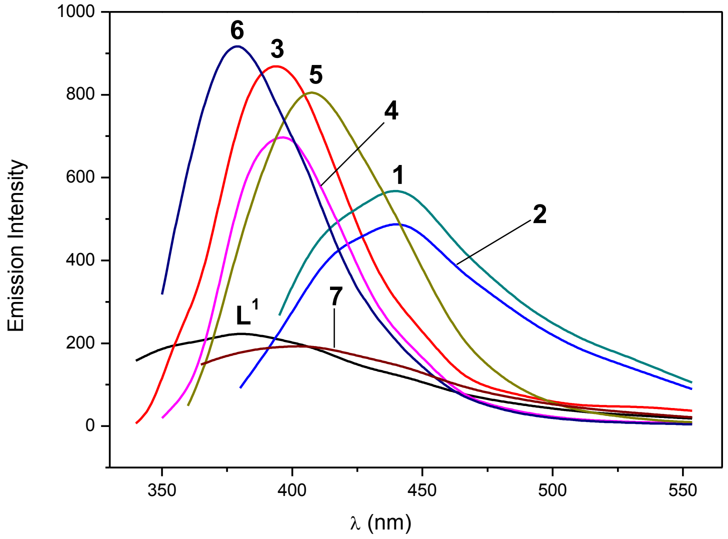

Figure S7. Photoluminescent properties of the L[1] and complexes **1-7** in DMSO. The peak of emission is 380 nm for L[1], 440 (**1**), 4440 (**2**), 395 (**3**), 396 (**4**), 408(**5**), 379(**6**), 402(**7**), respectively.

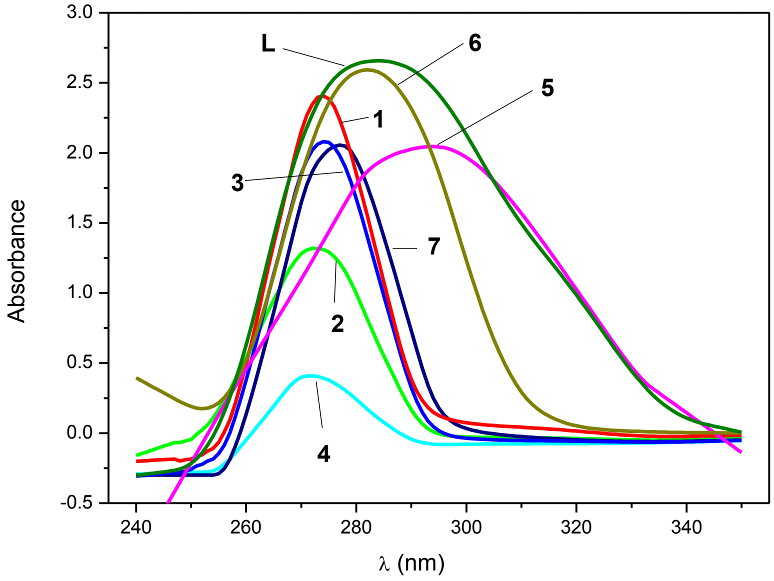

Figure S8. The UV-vis absorbance spectrum of the L[1] and complexes **1-7** in DMF.

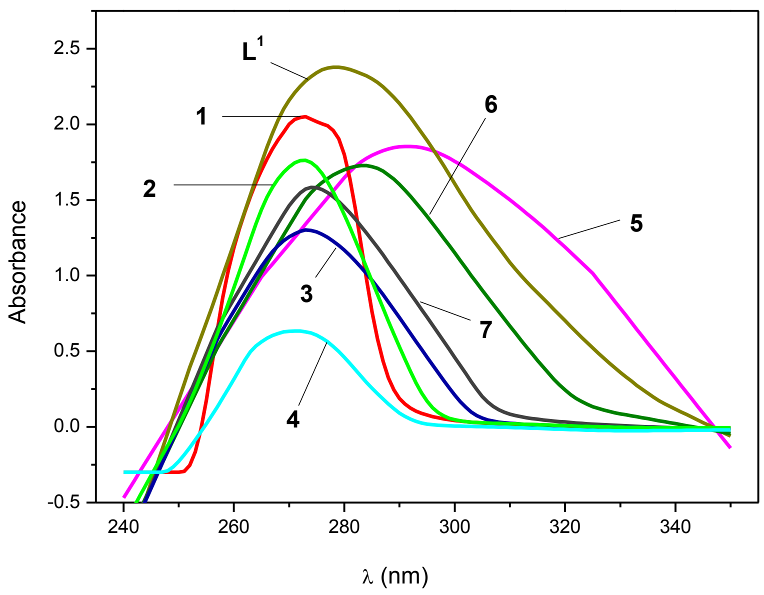

Figure S9. The UV-vis absorbance spectrum of the L[1] and complexes **1-7** in DMSO.
